# A longitudinal prospective cohort study investigating the association of premilking stimulation and teat-end shape on milking characteristics and teat tissue condition in dairy cows

**DOI:** 10.1186/s12917-019-1803-2

**Published:** 2019-02-12

**Authors:** Matthias Wieland, Jaclyn M. Melvin, Daryl V. Nydam, Paul D. Virkler

**Affiliations:** 1 Department of Population Medicine and Diagnostic Sciences, Cornell University, Ithaca, NY 14853 USA; 2 Department of Animal Science, Cornell University, Ithaca, NY 14853 USA; 3 Department of Population Medicine and Diagnostic Sciences, Cornell University, Ithaca, NY 14853 USA; 4 Department of Population Medicine and Diagnostic Sciences, Cornell University, Ithaca, NY 14853 USA

**Keywords:** Milking unit-on time, Two-minute milk, Time in low milk flow rate, Premilking udder preparation, Teat-end shape

## Abstract

**Background:**

Premilking udder preparation is essential for harvesting high-quality milk as gently, completely, and quickly as possible. The associations between characteristics such as teat-end shape and premilking stimulation on milking characteristics and machine milking-induced changes to the teat tissue condition have not been rigorously investigated. The primary objective was to investigate the interactive effects of manual premilking stimulation (i.e., preparation lag time) and teat-end shape on total milk yield, two-minute milk yield, milking unit-on time, and time in low milk flow rate. Our secondary objective was to study the association of manual premilking stimulation and changes to the teat tissue condition after machine milking (i.e., short-term changes). In a longitudinal prospective cohort study, 384 milking observations from 129 cows were analysed. Holstein cows were housed in sand-bedded free-stall pens, fed a total mixed ration, and milked 3 times a day. Cows were classified by teat-end shape into 1 of 3 categories: pointed, flat, or round. Individual cow milking characteristics were recorded with electronic on-farm milk meters. The duration of manual stimulation, preparation lag time, and presence of short-term changes were documented for each milking observation. General linear mixed models were used to study the interactive effects of preparation lag time and teat-end shape on milking characteristics.

**Results:**

There was an interaction between preparation lag time and teat-end shape for two-minute milk yield and time in low milk flow rate. The preparation lag time effect was modified by teat-end shape, while no interaction was observed for total milk yield or milking unit-on time. A generalized linear mixed model revealed that preparation lag time was associated with short-term changes in teat tissue condition, where the odds of short-term changes decreased as preparation lag time increased.

**Conclusions:**

In summary, cows with different teat-end shapes may require different premilking stimulation regimens. Increasing preparation lag time benefits teat tissue condition during machine milking. Further research is warranted to optimize individual premilking stimulation in dairy cows.

**Electronic supplementary material:**

The online version of this article (10.1186/s12917-019-1803-2) contains supplementary material, which is available to authorized users.

## Background

The ultimate objective of premilking udder preparation is to harvest high-quality milk from cows as gently, completely, and quickly as possible. In addition, it is valuable to monitor mammary health and thus animal well-being. Current recommendations for premilking udder preparation include teat sanitization (i.e., premilking teat disinfection, cleaning, and drying teats) and premilking stimulation [1]. Premilking stimulation is composed of some form of tactile stimulus (i.e., manual or mechanical stimulation) and milking unit attachment timing (i.e., preparation lag time). Premilking stimulation is essential for activating the milk-ejection reflex to harvest the alveolar milk, which represents approximately 80% of the udder’s milk volume [2–4]. Improper premilking stimulation reduces milk flow, which can result in increased vacuum load on the teat tissue [5, 6]. The premilking stimulation extent needed to achieve optimal milk flow is affected by stimulation technique [7, 8] and cow characteristics such as breed [9], stage of lactation [3, 9, 10], relative degree of udder filling, and milking interval [5, 11]. Although teat characteristics such as teat-end shape have been investigated in several studies on milk flow [12–14], their interactive effects with preparation lag time on milking characteristics have not been rigorously investigated.

Historically, most researchers have described the effects of premilking stimulation on milk yield, milking unit-on time, average milk flow rate, and peak milk flow rate [2, 7, 15]. Due to recent developments in on-farm milk meter technology, the ability to accurately measure milking characteristics has improved [10]. Among the many milking characteristics recorded by different milk meters from various dairy equipment manufacturers, two-minute milk yield and time in low milk flow rate are reported to be valuable measures of parlour performance including milking routine [16, 17]. Our study investigated the interactive effects of preparation lag time and teat-end shape on total milk yield, two-minute milk yield, milking unit-on time, and time in low milk flow rate in dairy cows milked 3 times daily. We hypothesized that the effect of preparation lag time on milking characteristics was modified by the effect of teat-end shape.

Most studies investigating premilking stimulation have concentrated on milking characteristics to improve parlour efficiency and profitability [18–20]. In contrast, information on the relationship between premilking stimulation and teat tissue condition after milking is scarce with no consensus [7, 21]. Albeit, the degree of machine milking-induced changes on teat tissue condition may be associated with the risk of new intramammary infections [22, 23]. Thus, reducing the risk and the degree of machine milking-induced changes in teat tissue condition may improve udder health and cow well-being. Our second objective, therefore, was to investigate the association between premilking stimulation and machine milking-induced changes to the teat tissue condition. We hypothesized that premilking stimulation was associated with machine milking-induced changes to the teat tissue condition.

## Methods

### Animals and housing

This study was conducted during October and November 2015 at the Teaching Dairy Barn of the College of Veterinary Medicine, Cornell University (Ithaca, NY). Holstein cows were housed in 2 free-stall pens with sand bedding and fed a total mixed ration consistent with National Research Council [24] requirements. Cow characteristics such as parity, stage of lactation (**DIM**, days in milk), and previous lactation 305-d mature equivalent milk yield were obtained from a dairy management software program (Dairy Comp 305, Valley Agricultural Software, Tulare, CA).

### Enrolment

The study population consisted of 150 lactating Holstein cows at the Teaching Dairy Barn of Cornell University. Cows (*n* = 129) without clinical mastitis for the previous 4 weeks and no udder abnormalities, such as nonlactating quarters or teat injuries, were eligible for enrolment. Data collection was subdivided into 3 intermittent days (3 consecutive Saturdays), evaluating a subset of cows to minimize the impact of data collection on milking routine. Data from each subset of cows were collected over 3 consecutive milking sessions.

### Teat-end shape

Teat-end shape on the quarter level was assessed prior to data collection with a digital voice recorder (ICD-UX533BLK, Sony, San Diego, CA) and classified into 1 of 3 categories: pointed, flat, or round. To investigate teat-end shape on cow level (**TES**), cows were classified into 3 groups based on the teat-end shape frequency of all 4 quarters as follows: cows with 2 or more pointed teats were classified as pointed, cows with 2 or more flat teats as flat, and cows with 3 or 4 round teats were classified as round. No cows were described to have 2 pointed and 2 flat teats or 2 round, 1 pointed, and 1 flat teat.

### Milking and premilking udder preparation

Cows were milked 3 times daily at 0400 (milking #1), 1100 (milking #2), and 1900 (milking #3) h in a 2 × 10 parallel milking parlour (P2100, DeLaval International AB, Tumba, Sweden). The system vacuum was 46 kPa with an average claw vacuum of 39 kPa during the peak milk flow period. The pulsator (EP100, 96,679,084, DeLaval International AB) was set at a pulsation rate of 60 cycles/min, a pulsation ratio of 65:35, and a side-to-side alternating pulsation (duration of pulsation phases: a-phase, 120; b-phase, 530; c-phase, 90; and d-phase, 260 ms). Milking clusters were removed automatically when milk flow decreased to below 1.3 kg/min (delay time: 0 s). The milking unit consisted of the milking cluster MC70, weighing 2.1 kg (DeLaval International AB), and the milking liner LS01 (LS01 SR 12 mm, 834,115,001, DeLaval International AB). The milking liner characteristics were as follows: mouthpiece bore diameter, 20.3 mm; barrel shape, round; barrel length, 113 mm; and barrel diameter at 75 mm, 21.8 mm. The long milk tube was silicone (90,843,501, DeLaval International AB) with an inner diameter of 19 mm. The milk line was installed 75 cm below cow standing level.

Milking routine was performed by 2 operators per session. Operators were composed of farm staff (*n* = 3) and students (*n* = 6) of the College of Veterinary Medicine, Cornell University. Premilking udder preparation was performed in sets of 5 cows and performed in 3 steps as follows: step 1 was to preclean the teats with a dry cloth towel and dip with an iodine-based teat dip (Udderdine110, Boumatic, Madison, WI, USA); step 2 was to forestrip all 4 teats then dry the teats with a separate clean cloth towel; and step 3 was to attach the milking unit. The time per task (precleaning, dipping, forestripping, drying time, and unit attachment) was recorded for each cow milking observation with a digital voice recorder (ICD-UX533BLK, Sony) by 2 investigators. Recorded time points (start and stop) per task were transferred to a spreadsheet (Microsoft Excel, 2013) using a comprehensive audio application program (Sound Organizer 4.1, Sony). Manual stimulation and preparation lag time durations were calculated. Manual stimulation time (**STIM**) was defined as the duration of forestripping and subsequent teat drying with a cloth towel, and preparation lag time (**LAG**) was defined as the time spent from the first teat stripping until the milking unit was attached.

### Milking characteristics

Total milk yield (kg; **TMY**), two-minute milk (kg; **2MIN**), milking unit-on time (s; **DUR**), time in low milk flow rate (s; **LMF**), and milking mode (manual versus automatic take-off) were assessed at each milking with electronic milk meters (MM27, DeLaval International AB) and recorded using the herd management system ALPRO (DeLaval International AB). Additional file 1: Table S1 provides definitions for reported milking characteristics.

### Machine milking-induced short-term changes to the teat tissue condition

Machine milking-induced short-term changes to the individual teat conditions were visually assessed by the same investigator within 60 s after unit detachment and based on the scoring system described by Hillerton et al. [25]. Key characteristics included skin colour changes, teat base condition, teat-end firmness, and teat orifice openness. Teat skin colour was evaluated as normal (score 0), red (score 1), or blue (score 2). Teat base condition showed no visible mark (score 0), visible mark present (score 1), or significant swelling (score 2). Teat-end consistency was scored soft (score 0), firm (score 1), or wedging present (score 2). The teat orifice was scored into 1 of 2 categories: opening < 2 mm (score 0) or opening ≥2 mm (score 1). All scores were documented with a digital voice recorder (ICD-UX533BLK, Sony) and subsequently transferred to a spreadsheet (Microsoft Excel, 2013). For subsequent analysis, machine milking-induced short-term changes to the teat condition were dichotomized as follows. A short-term change was present if the teat base score condition was 2, or the teat-end consistency score was ≥1, or the teat orifice score was 1. Short-term changes were considered absent otherwise. Pigmentation was noted in 84/516 (16.3%) of the teats. Because this impeded consistent evaluation, teat skin colour was excluded from subsequent analyses.

### Analytical approach

Prior to statistical analyses, the data from 387 cow-milking observations of 129 cows were investigated for missing and erroneous values, and 1 milking observation each from 3 cows were excluded because of erroneous values (average milk flow rate = 0 kg/min). Statistical analyses were performed using R Statistical Software (R Core Team, [26]).

### Baseline characteristics

Descriptive statistics of the previous lactation 305-d mature equivalent milk yield, TMY, 2MIN, DUR, LMF, STIM, and LAG are presented as the mean and standard deviation, median, minimum value, and maximum value. Stage of lactation (DIM) was stratified into 3 categories (early lactation, ≤ 100; mid-lactation, 101–200; and late lactation, > 200 DIM), and parity was classified into 3 categories (1st, 2nd, and ≥ 3rd lactation). Frequency distributions for parity, DIM, and TES are presented as absolute values and percentage.

### Milking characteristics

Cow milking observation was the unit of analysis, and the continuous variables, TMY, 2MIN, DUR, and LMF, were the outcomes of interest. A separate general linear mixed model was fitted for each dependent variable (TMY, 2MIN, DUR, and LMF) using the ‘nlme’ package [27]. To account for clustering of milking session within cow, cow was included as a random effect. To model the within-cow covariance of repeated measurements, 3 covariance structures (compound symmetry, autoregressive order 1, and unstructured) were tested for each dependent variable investigated. The covariance structure with the smallest Akaike’s information criterion was selected. F-tests for Type-III analysis of variance were calculated using the ‘car’ package [28]. The initial model included the independent variables: milking session, parity, DIM, TES, STIM, LAG, and the interaction term between TES and LAG. For the dependent variables 2MIN, DUR, and LMF, TMY was also included as an independent variable. Collinearity among eligible variables was assessed by calculating the variance inflation factor from the initial models. A variance inflation factor > 5 was considered to indicate multicollinearity. No collinearity was observed among the variables: milking session, parity, DIM, TMY, TES, STIM, or LAG. Backward stepwise selection was performed until each independent variable had a *p*-value of ≤ 0.05. Confounding effects were monitored by observing regression coefficient changes. Variables that modified regression coefficients by > 20% were considered confounding factors. No confounding was observed. For the final models, homoscedasticity and residual normality assumptions were assessed by plotting residuals versus corresponding predicted values and examining residual quantile-quantile plots. To satisfy these assumptions, data for the dependent variable LMF were log transformed. Resulting coefficient and least squares estimates were consequently back-transformed and presented as the geometric mean and 95% CI. Least squares means (LSM) ± SE and LSM (95% CI) were calculated using the ‘lsmeans’ package [29]. Results were averaged over all variable levels included in the final model. Tukey-Kramer’s post hoc test was used to control for experiment-wise error rate for comparing means across different categorical variable levels. Least squares means ± SE and LSM (95% CI) were calculated with the ‘at’ function in the ‘lsmeans’ package [29] as follows: 2MIN for a hypothetical milking observation during milking #1 of a cow with a 15-kg TMY; pointed, flat, and round TES; and a LAG of 60 and 90 s, respectively; LMF for a hypothetical milking observation during milking #1 of a cow in 2nd lactation with a 15-kg TMY; pointed, flat, and round TES; and a LAG of 60 and 90 s, respectively.

### Machine milking-induced short-term changes to the teat tissue condition

Cow milking observation was the unit of analysis, and the dichotomous variable, machine milking-induced short-term changes to the teat condition on cow level (**STC**), was the outcome of interest. Machine milking-induced short-term changes to the teat condition on cow level were considered present if 1 or more teats showed short-term changes as defined above, and absent otherwise. A generalized linear mixed model with a logit link and a binomial distribution was fitted with the ‘lme4’ package to determine the association between the dependent variable, STC, and the independent variables, milking session, parity, DIM, TES, TMY, STIM, and LAG [30]. To account for within-cow clustering between milking sessions, cow was included as random effect. Univariable associations were tested between the dependent and all independent variables. All variables with a *p*-value ≤ 0.20 in this step were considered for inclusion in the initial model. Collinearity among eligible variables was assessed by Pearson’s correlation for continuous variables and Spearman’s correlation for categorical variables. No collinearity was observed among the eligible variables, milking session, parity, DIM, TMY, TES, and LAG (correlation coefficients ≤ |0.41|). Backward stepwise selection was performed until each independent variable had a *p*-value of ≤ 0.05. Type-III Wald chi-square tests were calculated using the ‘car’ package [28]. Two-way interactions between remaining variables were investigated and retained in the model if *p*-value ≤ 0.05. Pearson goodness-of-fit statistic of a logistic regression model, including the same fixed but not random effects, was used to indirectly assess the final model fit. The adjusted probabilities (95% CI) of STC for a hypothetical milking observation during milking #1 of a late lactation cow, and a LAG of 60 and 90 s was calculated using the ‘AICcmodavg’ package [31].

## Results

### Description of study population

A total of 384 milking observations from 129 cows were included in the final analysis. The mean ± SD (range) for previous lactation 305-d mature equivalent milk yield was 13,464 ± 2828 (5838-19,296) kg. The mean ± SD DIM was 152 ± 105 and ranged from 11 to 512 d. Parity was distributed as follows: 52 (40.3%) were in the 1st, 27 (20.9%) were in the 2nd, and 50 (38.8%) cows were in the 3rd or greater lactation. Teat-end shape was distributed as follows: pointed, 18 (14.0%); flat, 11 (8.5%); and round, 100 (77.5%) cows. Two (0.5%) milking observations were performed on manual milking mode. The overall mean ± SD values for TMY, 2MIN, DUR, and LMF were 13.6 ± 3.9 kg; 6.4 ± 2.3 kg; 244 ± 79 s; and 21 ± 40 s, respectively. Descriptive statistics for TMY, 2MIN, DUR, and LMF, stratified by parity, DIM, TES, and milking session, respectively, are provided in Table 1. The overall mean ± SD values for STIM and LAG were 11 ± 4 and 74 ± 19 s, respectively. Short-term changes to the teat condition on cow level (STC) were observed in 38/54 (70%), 12/33 (36%), and 172/297 (58%) milking observations in cows with pointed, flat, and round TES, respectively. Additional file 2: Table S2 depicts descriptive statistics for STIM, LAG, and STC, stratified by TES.

**Table 1:** Descriptive statistics of total milk yield (TMY, kg), two-minute milk yield (2MIN, kg), milking unit-on time (DUR, s), and time in low milk flow rate (LMF, s), stratified by parity, stage of lactation (DIM), teat-end shape (TES), and milking session in 384 milking observations of 129 cows

		TMY				2MIN				DUR				LMF			
Item	n^a^	Mean	SD	Median	Range	Mean	SD	Median	Range	Mean	SD	Median	Range	Mean	SD	Median	Range
Parity																	
1st	52	12.3	2.3	12.2	5.9–18.5	6.4	2.1	6.3	0.5–11.6	215	61	208	105–427	15	13	11	4–127
2nd	27	15.0	3.7	14.9	4.7–24.9	6.4	2.6	6.3	0.0–12.2	261	68	251	163–455	24	56	11	5–427
≥3rd	50	14.3	4.9	14.7	2.4–26.2	6.3	2.3	6.4	0.0–12.3	267	90	249	128–546	26	46	12	5–306
DIM																	
≤ 100	51	14.4	3.8	14.2	5.9–26.2	6.4	2.4	6.3	0.0–12.3	253	83	243	105–546	16	23	12	5–225
101–200	40	14.0	3.2	13.7	6.0–23.1	6.9	2.1	7.0	1.4–12.2	237	75	219	125–455	15	14	11	4–115
> 200	38	12.2	4.4	11.8	2.4–24.4	5.7	2.1	5.7	0.0–9.6	240	76	230	128–438	33	65	12	6–427
TES																	
Pointed	18	14.5	4.6	14.5	5.9–26.2	5.2	1.9	5.3	0.0–10.6	289	101	272	105–527	25	59	13	7–427
Flat	11	13.4	3.8	13.5	6.0–21.7	6.9	2.2	6.8	2.5–12.0	223	68	218	128–393	16	15	11	6–63
Round	100	13.5	3.8	13.2	2.4–24.9	6.5	2.3	6.7	0.0–12.3	238	72	229	125–546	20	37	11	4–306
Milking session																	
Milking #1	–	15.5	4.2	15.1	2.4–26.2	6.6	2.4	6.7	0.0–12.3	270	81	261	105–546	20	47	11	6–427
Milking #2	–	11.4	3.1	11.3	2.4–19.5	5.7	2.2	5.7	0.0–11.1	218	66	207	119–523	28	45	13	6–306
Milking #3	–	14.0	3.3	13.7	5.1–23.8	6.8	2.2	6.8	0.5–12.2	244	79	234	112–496	15	20	11	4–179

^a^Number of cows for each category

### Milking characteristics

The interactive effects of LAG and TES on milking characteristics were evaluated based on the 4 milking characteristics, TMY, 2MIN, DUR, and LMF. General linear mixed model results are provided in Additional file 3: Tables S3-S6 and described separately below.

### Total milk yield

The final multivariable general linear mixed model for TMY contained the fixed effects: milking session (*p* <  0.001), parity (*p* <  0.001), and DIM (*p* < 0.001). Controlling for all other covariates, the means (LSM ± SE) for TMY were 15.7 ± 0.3, 11.6 ± 0.3, and 14.1 ± 0.3 kg during milking sessions #1, #2, and #3, respectively, and were different between all milking sessions (*p* < 0.001). The means (LSM ± SE) for TMY were 12.1 ± 0.4, 15.0 ± 0.5, and 14.4 ± 0.4 kg for cows in 1st, 2nd, and 3rd or greater lactations, respectively. Tukey-Kramer’s post hoc test revealed differences between primiparous and 2nd lactation animals (*p* < 0.001), as well as between primiparous and cows in 3rd or greater lactation (*p* < 0.001). Total milk yields (LSM ± SE) were 14.7 ± 0.4, 14.4 ± 0.5, and 12.4 ± 0.5 kg in early, mid, and late lactation cows, respectively. Tukey-Kramer’s post hoc test revealed differences between early and late lactation cows (*p* < 0.001) and mid and late lactation cows (*p* < 0.001) (Additional file 3: Table S3).

### Two-minute milk yield

**Fig. 1:**
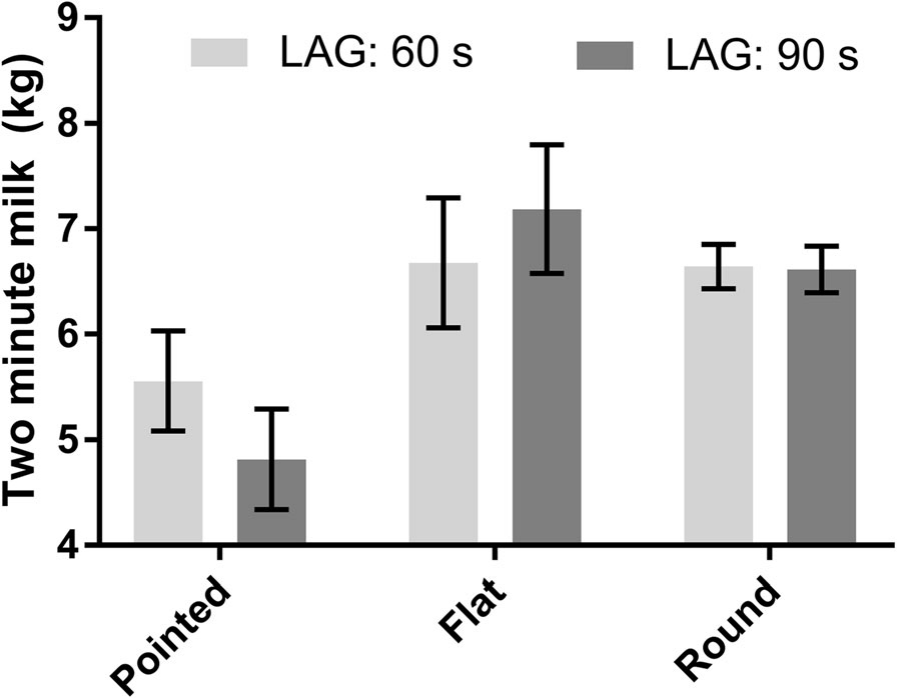
Two-minute milk yield for a hypothetical milking observation during milking #1 of a cow with a total milk yield of 15 kg; pointed, flat, and round teat-end shape; and a preparation lag time (LAG) of 60 and 90 s, respectively. The results are presented as least squares means (kg) from a general linear mixed model. Error bars show standard errors

The final multivariable general linear mixed model for 2MIN contained the following fixed effects: milking session (*p* < 0.001), TMY (*p* < 0.001), TES (*p* = 0.6), LAG (*p* = 0.8), and the interaction term between TES and LAG (*p* = 0.003) (Additional file 3: Table S4). Two-minute milk yields (LSM ± SE) for a hypothetical milking observation during milking #1 of a cow with a TMY of 15 kg were 5.6 ± 0.5, 6.7 ± 0.6, and 6.6 ± 0.2 kg for pointed, flat, and round TES, respectively for a LAG of 60 s; and 4.8 ± 0.5, 7.2 ± 0.6, and 6.6 ± 0.2 kg for pointed, flat, and round TES, respectively for a LAG of 90 s (Fig. 1).

### Milking unit-on time

The final multivariable general linear mixed model for DUR contained the following fixed effects: milking session (*p* = 0.02), TMY (*p* < 0.001), parity (*p* = 0.03), and TES (*p* = 0.02). Controlling for all other variables included in the model, the DUR means (LSM ± SE) were 255 ± 8, 241 ± 8, and 243 ± 8 s, during milkings #1, #2, and #3, respectively. Tukey-Kramer’s post hoc test revealed differences between milking #1 and #3 (*p* = 0.02), but no differences between milking #1 and #2 (*p* = 0.07) and milking #2 and #3 (*p* = 0.92), respectively. A 1-kg increase in TMY increased DUR by 10 ± 1 s. The DUR means (LSM ± SE) were 277 ± 13, 220 ± 17, and 241 ± 6 s in cows with pointed, flat, and round TES, and these differed between cows with pointed and flat TES (*p* = 0.03) and between cows with pointed and round TES (*p* = 0.04), respectively. Milking unit-on time was 230 ± 11 s in primiparous cows, 248 ± 12 s in cows in 2nd lactation, and 261 ± 9 s in cows in 3rd or greater lactation and was different between primiparous animals and cows in 3rd or greater lactation (*p* = 0.03) (Additional file 3: Table S5).

### Time in low milk flow rate

**Fig. 2:**
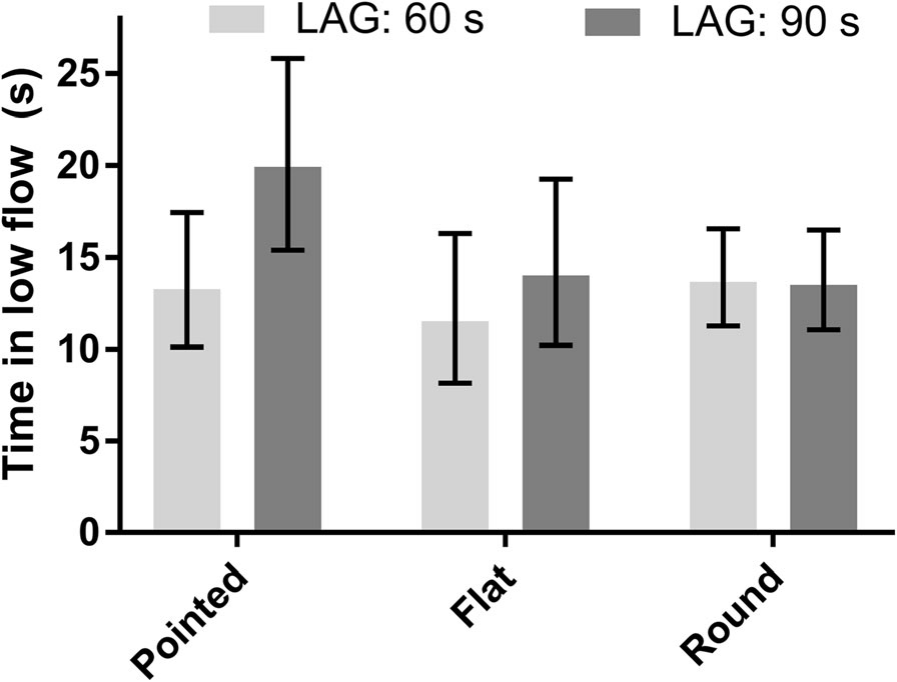
Time in low milk flow rate for a hypothetical milking observation during milking #1 of a cow in 2nd lactation, a total milk yield of 15 kg; pointed, flat, and round teat-end shape; and a preparation lag time (LAG) of 60 and 90 s, respectively. The results are presented as least squares means (s) from a general linear mixed model. Error bars show 95% CI

The final multivariable general linear mixed model for LMF contained the following fixed effects: milking session (*p* < 0.001), TMY (*p* < 0.001), parity (*p* = 0.004), TES (*p* = 0.007), LAG (*p* = 0.8), and the interaction term between TES and LAG (*p* < 0.001) (Additional file 3: Table S6). Time in low milk flow rates (LSM, 95% CI) for a hypothetical milking observation during milking #1 of a cow in 2nd lactation with a TMY of 15 kg; were 13, 10–17; 12, 8–16; and 14, 11–17 s for pointed, flat, and round TES, respectively for a LAG of 60 s; and 20, 15–26; 14, 10–19; and 13, 11–16 s for pointed, flat, and round TES, respectively for a LAG of 90 s (Fig. 2).

### Machine milking-induced short-term changes to the teat tissue condition

Table 2 depicts unadjusted regression coefficients, SE, unadjusted odds ratios (**OR**), and 95% CI for independent variables tested for the association with STC from univariable generalized linear mixed models. The final multivariable generalized linear mixed model for STC presence or absence contained the following fixed effects: milking session (*p* < 0.001), DIM (*p* = 0.005), and LAG (*p* = 0.005), while none of the tested interactions remained in the model (Table 3). The adjusted probability (95% CI) of STC for a hypothetical milking observation during milking #1 of a late lactation cow and a LAG of 60 and 90 s was 60% (37–80%) and 40% (20–64%), respectively.

**Table 2:** Unadjusted regression coefficients, SE, unadjusted odds ratios, and 95% CI for independent variables tested for association with machine milking-induced short-term changes to the teat condition from univariable generalized linear mixed models from 384 milking observations of 129 cows

Explanatory variable	β^a^ (SE)	unadj.OR^b^ (95% CI)	*p*
Milking session			0.001
Milking #1	0.30 (0.35)	1.34 (0.68–2.64)	
Milking #2	−0.97 (0.34)	0.38 (0.19–0.75)	
Milking #3	-Referent-		
Parity			0.06
1st	1.10 (0.51)	3.01 (1.10–8.24)	
2nd	0.01 (0.58)	1.02 (0.33–3.17)	
≥ 3rd	-Referent-		
DIM^c^			0.003
≤ 100	1.84 (0.55)	6.28 (2.12–18.59)	
101–200	0.72 (0.55)	2.05 (0.70–5.99)	
> 200	-Referent-		
TES^d^			0.06
Pointed	0.90 (0.65)	2.45 (0.68–8.75)	
Flat	−1.41 (0.79)	0.24 (0.05–1.15)	
Round	-Referent-		
TMY^e^	0.12 (0.05)	1.13 (1.02–1.25)	0.02
STIM^f^	0.02 (0.37)	1.02 (0.49–2.11)	0.95
LAG^g^	−0.15 (0.07)	0.86 (0.74–1.01)	0.06

^a^Unadjusted linear regression coefficient

^b^Unadjusted odds ratio

^c^Stage of lactation (days in milk)

^d^Teat-end shape. Classified as follows: pointed = 2 or more pointed teats, flat = 2 or more flat teats, round = 3 or 4 round teats

^e^TMY = Total milk yield (kg)

^f^Stimulation time (s)

^g^Preparation lag time (s), time between first teat stripping and milking unit attachment

^e-g^Odds ratios are for each 1-unit increase, TMY: 1 kg; STIM: 10 s; LAG: 10 s

**Table 3:** Multivariable generalized linear mixed model describing the factors associated with presence or absence of milking machine-induced short-term change to teat tissue condition from 384 milking observations of 129 cows

Item	ß^a^ (SE)	*p*	aOR^b^ (95% CI)
Intercept	2.14 (0.97)	0.03	–
Milking session		< 0.001	
Milking #1	−0.07 (0.38)		0.92 (0.44–1.93)
Milking #2	−1.48 (0.41)		0.23 (0.10–0.51)
Milking #3	-Referent-		
DIM^c^		0.005	
≤ 100	2.00 (0.63)		7.40 (2.17–25.26)
101–200	0.75 (0.61)		2.11 (0.63–7.01)
> 200	-Referent-		
LAG^d^	−0.27 (0.10)	0.005	0.76 (0.63–0.92)

^a^Linear regression coefficient

^b^Adjusted odds ratio

^c^Stage of lactation (days in milk)

^d^Preparation lag time (s, 1-unit = 10 s); time between first teat stripping and milking unit attachment

## Discussion

This study investigated the interactive effects of LAG and TES on milking characteristics (TMY, 2MIN, DUR, and LMF). We observed an interaction between TES and LAG for 2MIN and LMF, but no interaction for TMY and DUR. The LAG effect was modified by TES, as an increased LAG yielded an increased 2MIN in cows with flat TES, while it had no meaningful effect in cows with round TES, and it decreased the 2MIN in cows with pointed TES. In addition, increased LAG yielded higher LMF in cows with pointed and flat TES, but resulted in a slightly decreased LMF in cows with round TES; thus, cows with different TES responded differently to LAG. This suggests that cows with different TES may require different premilking stimulation regimens to achieve optimal milk flow. Our study adds to the existing literature indicating that cows with different characteristics (i.e., udder filling and stage of lactation) require different preparation lag times to achieve optimal milk let-down [5, 10, 18]. Addressing these requirements in premilking stimulation may improve parlour efficiency and alleviate the potentially harmful effects of machine milking on teat tissue condition in dairy cows.

The lack of association between premilking stimulation and TMY is consistent with some studies [15, 32, 33], but contrasts with other studies that found a positive association between premilking stimulation and milk production [8, 9, 34].

Teat-end shape was associated with DUR, as cows with flat TES milked fastest, while those with pointed TES milked slowest. Our findings indicated that milkability differed among cows with different TES, supporting descriptions by other investigators that cows with flat TES had the largest 2MIN [14, 35]. Increased milking speed has been a breeding objective for increased milking efficiency and parlour throughput in recent years [36, 37]. As suggested by Guarín and Ruegg [38], this may have influenced teat characteristics such as TES. Possible factors associated with TES that may explain differences in cow milkability are teat canal length [12] and diameter [39]. In contrast to previous studies [2, 33, 40], premilking stimulation did not influence DUR in this study. Discrepancies in milking frequency and automatic cluster removal thresholds could account for the differences observed between those studies and this one. Differences in milking characteristics among milking sessions were likely attributed to differences in milking intervals between sessions, as well as differences in milking routine including cow handling and premilking stimulation (type of forestripping) among different operators.

Several factors may explain the interactive effects of TES and LAG on milking characteristics. Possible explanations include different dimensions in anatomical structures such as teat canal and gland cistern, which may act in concert with milking machine settings and equipment (i.e., milking liner) yielding different responses to premilking stimulation among cows with different TES. Another variable that may explain this is the adrenoceptor pattern difference (beta-2:alpha-2-adrenoceptor ratio), which may have led to differences in the premilking stimulation response, and thus milkability among cows with different TES. However, although these anatomical and physiological characteristics have been reported to be associated with milkability by previous investigators [12, 41, 42], these hypotheses remain to be tested. Therefore, this work must be extended to further understand the differences in milkability among cows with different characteristics and determine the potential of the individual premilking stimulation regimen as a possible method of improving milking efficiency, teat tissue condition, and cow well-being.

Our study also investigated the association between premilking stimulation and STC. Our results show that increased LAG decreased STC probability, while no association was seen between STIM and STC. This contrasts with results reported by Vetter et al. [7] who found no differences in teat tissue condition as assessed by ultrasonography when comparing 2 different premilking udder stimulation regimens. Several reasons may explain the benefits of LAG on teat tissue condition after machine milking.

One reason could be that LAG may affect teat diameter. Teat diameter has been shown to increase before milking due to milk accumulating in the teat cistern [43]. To form a seal between the teat and milking liner barrel, teats must be a few mm wider than the liner barrel diameter [44]. Therefore, the increased LAG in our study may have resulted in an increased teat diameter, yielding a better seal between the teat and the liner barrel. This could have lowered the mouthpiece chamber vacuum [45], thereby decreasing teat congestion and oedema, as outlined by Penry et al. [46]. Another possible explanation could have been that LAG affected the milk flow rate. Multiple studies demonstrated that preparation lag time positively affects continuous milk flow and decreases bimodal milk let-down [8, 10, 18], which is reported to increase vacuum load resulting in teat congestion and oedema [2, 5].

Although we intended to best represent current milking practices, our study had some limitations that must be considered. To reduce inadvertent variability, we excluded cows with a history of mastitis during the 4 weeks prior to enrolment as well as cows with a nonlactating quarter. This likely introduced a selection bias and decreased the generalizability of our results. Another limitation may have been the subjective assessment of TES and STC, which may have yielded information bias. Another limitation is that the study was observational and has been conducted on a single farm during one season (i.e., fall); thus, the results should be interpreted cautiously. These results should be confirmed in a controlled experiment investigating the effect of different premilking stimulation regimens in cows with different TES from several dairy farms considering the effect of climate and season across different regions, as well as different management systems (e.g., housing and milking routine).

## Conclusions

In conclusion, LAG and TES had an interactive effect on 2MIN and LMF, indicating that cows with different TES respond differently to premilking stimulation. In contrast, premilking stimulation was not associated with TMY or DUR. Preparation lag time was associated with STC probability, which decreased as the LAG increased. Further studies investigating individual milk flow curves among cows with different TES are warranted to better understand the interactive effects of cow characteristics, machine milking, and teat and udder health. The results from these studies may increase milking efficiency and alleviate potentially detrimental effects from machine milking on teat tissue condition in dairy cows. Individual premilking stimulation may be applied to automatic milking systems, which would allow specific milking routines for individual cows and teats.

## Additional files

Additional file 1: **Tabel S1.** Definitions of milking characteristics recorded with the electronic milk meters. (DOCX 12 kb)

Additional file 2: **Tabel S2.** Descriptive statistics of manual stimulation time (s), preparation lag time (s), and short-term changes to the teat condition on cow level (n, %), stratified by teat-end shape from 384 milking observations of 129 cows. (DOCX 12 kb)

Additional file 3: **Tabel S3-S6.** General linear mixed model results describing the factors associated with total milk yield, two-minute milk yield, milking unit-on time, and time in low milk flow rate from 384 milking observations of 129 cows. (DOCX 19 kb)

Additional file 4: Raw data used for the statistical analyses in this study. (XLSX 34 kb)

## Abbreviations

2MIN: Two-minute milk yield
DIM: Days in milk
DUR: Milking unit-on time
LAG: Preparation lag time
LMF: Time in low milk flow rate
LSM: Least squares means
SD: Standard deviation
SE: Standard error
STC: Milking machine-induced short-term changes in teat tissue condition
STIM: Manual stimulation time
TES: Teat-end shape
TMY: Total milk yield

## References

[1] Recommended milking procedures. National Mastitis Council, Inc., Madison, WI. http://www.nmconline.org/wp-content/uploads/2016/09/Recommended-Milking-Procedures.pdf. Accessed 12 Oct 2017.

[2] Bruckmaier RM, Blum JW (1996). Simultaneous recording of oxytocin release, milk ejection and milk flow during milking of dairy cows with and without prestimulation. J Dairy Res.

[3] Bruckmaier RM, Blum JW (1998). Oxytocin release and milk removal in ruminants. J Dairy Sci.

[4] Pfeilsticker HU, Bruckmaier RM, Blum JW (1996). Cisternal milk in the dairy cow during lactation and after preceding teat stimulation. J Dairy Res.

[5] Bruckmaier RM, Hilger M (2001). Milk ejection in dairy cows at different degrees of udder filling. J Dairy Res.

[6] Bruckmaier RM, Rothenanger E, Blum JW (1995). Milking characteristics in dairy cows of different breeds from different farms and during the course of lactation. J Anim Breed Genet.

[7] Vetter A, van Dorland HA, Youssef M, Bruckmaier RM (2014). Effects of a latency period between pre-stimulation and teat cup attachment and periodic vacuum reduction on milking characteristics and teat condition in dairy cows. J Dairy Res.

[8] Watters RD, Bruckmaier RM, Crawford HM, Schuring N, Schukken YH, Galton DM (2015). The effect of manual and mechanical stimulation on oxytocin release and milking characteristics in Holstein cows milked 3 times daily. J Dairy Sci.

[9] Rasmussen MD, Frimer ES, Galton DM, Petersson LG (1992). The influence of Premilking teat preparation and attachment delay on Milk yield and milking performance. J Dairy Sci.

[10] Watters RD, Schuring N, Erb HN, Schukken YH, Galton DM (2012). The effect of premilking udder preparation on Holstein cows milked 3 times daily. J Dairy Sci.

[11] Weiss D, Dzidic A, Bruckmaier RM (2003). Effect of stimulation intensity on oxytocin release before, during and after machine milking. J Dairy Res.

[12] Grindal RJ, Walton AW, Hillerton JE (1991). Influence of milk flow rate and streak canal length on new intramammary infection in dairy cows. J Dairy Res.

[13] Weiss D, Weinfurtner M, Bruckmaier RM (2004). Teat anatomy and its relationship with quarter and udder Milk flow characteristics in dairy cows. J Dairy Sci.

[14] Seykora AJ, McDaniel BT (1985). Heritabilities of teat traits and their relationships with Milk yield, somatic cell count, and percent two-minute Milk. J Dairy Sci.

[15] Wagner AM, Ruegg PL (2002). The effect of manual forestripping on milking performance of Holstein dairy cows. J Dairy Sci.

[16] Treichler BK, Reid DA (2013). Review of parlor summaries from 3x herds in the United States. Proceedings of the 52nd National Mastitis Council Annual Meeting, San Diego, CA.

[17] Reid DA, Stewart S (2007). Using electronic data to monitor and improve parlor performance. Proceedings of the 46th National Mastitis Council Annual Meeting, San Antonio, TX.

[18] Weiss D, Bruckmaier RM (2005). Optimization of individual prestimulation in dairy cows. J Dairy Sci.

[19] Smith JF, Dhuyvetter KC, VanBaale MJ, Armstrong DV, Harner J (2005). Managing the milking parlor: An economic consideration of profitability. Proceedings of the 44th National Mastitis Council Annual Meeting, Orlando, FL.

[20] Gorewit RC, Gassman KB (1985). Effects of duration of udder stimulation on milking dynamics and oxytocin release. J Dairy Sci.

[21] Hamann J (1992). Physio-pathological aspects of machine milking. International symposium on bovine mastitis. Milan, Italy.

[22] Zecconi A, Hamann J, Bronzo V, Ruffo G (1992). Machine-induced teat tissue reactions and infection risk in a dairy herd free from contagious mastitis pathogens. J Dairy Res.

[23] Zecconi A, Bronzo V, Piccinini R, Moroni P, Ruffo G (1996). Field study on the relationship between teat thickness changes and intramammary infections. J Dairy Res.

[24] National Research Council (2001). Nutrient requirements of dairy cattle: seventh revised edition, 2001. The.

[25] Hillerton JE, Ohnstad I, Baines JR, Leach KA (2000). Changes in cow teat tissue created by two types of milking cluster. J Dairy Res.

[26] R Core Team: R: A Language and Environment for Statistical Computing. R Foundation for Statistical Computing, Vienna: 2016. https://www.r-project.org/. Accessed 12 Oct 2017.

[27] Pinheiro J, Bates D, DebRoy S, Sarkar D, Team RC. Nlme: linear and nonlinear mixed effects models. R package version. 2016:31–128 http://CRAN.R-project.org/package=nlme.

[28] Fox J, Weisberg S, An R. Companion to applied regression. 2nd ed. Thousand Oaks, CA: Sage; 2011.

[29] Lenth RV (2016). Least-squares means: the R package lsmeans. J Stat Softw.

[30] Bates D, Maechler M, Bolker B, Walker S (2015). Fitting linear mixed-effects models using lme4. J Stat Softw.

[31] Mazerolle MJ. AICcmodavg: model selection and multimodel inference based on (Q)AIC(c). R package version. 2016:21–0 https://cran.r-project.org/package=AICcmodavg.

[32] Edwards JP, Jago JG, Lopez-Villalobos N (2013). Short-term application of prestimulation and increased automatic cluster remover threshold affect milking characteristics of grazing dairy cows in late lactation. J Dairy Sci.

[33] Kaskous S, Bruckmaier RM (2011). Best combination of pre-stimulation and latency period duration before cluster attachment for efficient oxytocin release and milk ejection in cows with low to high udder-filling levels. J Dairy Res.

[34] Merrill WG, Sagi R, Petersson LG, Bui TV, Erb HN, Galton DM, Gates R (1987). Effects of premilking stimulation on complete lactation milk yield and milking performance. J Dairy Sci.

[35] Hodgson AS, Murdock FR (1980). Effect of teat-end shape on milking rate and udder health. J Dairy Sci.

[36] Byrne TJ, Santos BF, Amer PR, Martin-Collado D, Pryce JE, Axford M (2016). New breeding objectives and selection indices for the Australian dairy industry. J Dairy Sci.

[37] Govignon-Gion A, Dassonneville R, Baloche G, Ducrocq V (2016). Multiple trait genetic evaluation of clinical mastitis in three dairy cattle breeds. Animal.

[38] Guarín JF, Ruegg PL (2016). Short communication: pre- and postmilking anatomical characteristics of teats and their associations with risk of clinical mastitis in dairy cows. J Dairy Sci.

[39] Rathore AK, Sheldrake RF (1977). Teat orifice stretchability associated with teat diameter gradient and milk yield in lactating cows. Animal Sci.

[40] Schukken YH, Petersson LG, Nydam DV, Baker DE (2005). Team F. Using Milk flow curves to evaluate milking procedures and Milk equipment. Proceedings of the 44th National Mastitis Council Annual Meeting, Orlando, FL.

[41] Roets E, Vandeputte-Van Messom G, Peeters G (1986). Relationship between milkability and adrenoceptor concentrations in teat tissue in primiparous cows. J Dairy Sci.

[42] Roets E, Vandeputte-Van Messom G, Burvenich C, Peeters G (1989). Relationship between numbers of alpha 2- and beta 2-adrenoceptors in teat tissue and blood cells and milkability of primiparous cows. J Dairy Sci.

[43] Kuchler K. [Investigation of the effects of milking on the teat tissue and the teat blood flow using ultrasonographic scanning and color angiography]. Thesis. 2011. Ludwig-Maximilians-Universität. Munich, Germany.

[44] Rasmussen MD (1997). The relationship between mouthpiece vacuum, teat condition, and udder health. Proceedings of the 36th National Mastitis Council Annual Meeting, Madison, WI.

[45] Borkhus M, Rønningen O (2003). Factors affecting mouthpiece chamber vacuum in machine milking. J Dairy Res.

[46] Penry JF, Upton J, Mein GA, Rasmussen MD, Ohnstad I, Thompson PD, Reinemann DJ (2017). Estimating teat canal cross-sectional area to determine the effects of teat-end and mouthpiece chamber vacuum on teat congestion. J Dairy Sci.

