# Supplementary material for: A longitudinal prospective cohort study investigating the association of premilking stimulation and teat-end shape on milking characteristics and teat tissue condition in dairy cows
Source: BMC Vet Res. 2019 Feb 12;15:58. doi: 10.1186/s12917-019-1803-2 (PMC6373114; doi:10.1186/s12917-019-1803-2)
Supplement: Supplementary file 1 — Tabel S1. Definitions of milking characteristics recorded with the electronic milk meters. (DOCX 12 kb) [file 12917_2019_1803_MOESM1_ESM.docx]

**Supplementary Table 1.** Definitions of total milk yield and milking characteristics recorded with the automatic milk meter (MM27, DeLaval International AB, Tumba, Sweden).

| Item | Abbreviation | Definition |
| --- | --- | --- |
| Total milk yield (kg) | TMY | Milk yield recorded from start of milking^1^ to unit detachment |
| Two-minute milk yield (kg) | 2MIN | Milk yield harvested within the first 2 minutes after start of milking^1^ |
| Milking unit-on time (s) | DUR | Time recorded from start of milking^1^ to unit detachment |
| Time in low milk flow rate (s) | LMF | Seconds below 1 kg/min milk flow rate recorded throughout entire milking (i.e., start of milking to unit detachment) |

^1^Start of milking: Start of milking procedure as recorded by the milking point controller (MPC680, DeLaval International AB, Tumba, Sweden) induced by the push of start button.
