# Supplementary material for: A longitudinal prospective cohort study investigating the association of premilking stimulation and teat-end shape on milking characteristics and teat tissue condition in dairy cows
Source: BMC Vet Res. 2019 Feb 12;15:58. doi: 10.1186/s12917-019-1803-2 (PMC6373114; doi:10.1186/s12917-019-1803-2)
Supplement: Supplementary file 2 — Tabel S2. Descriptive statistics of manual stimulation time (s), preparation lag time (s), and short-term changes to the teat condition on cow level (n, %), stratified by teat-end shape from 384 milking observations of 129 cows. (DOCX 12 kb) [file 12917_2019_1803_MOESM2_ESM.docx]

**Supplementary Table 2.** Descriptive statistics of manual stimulation time (STIM, s), preparation lag time (LAG, s), and short-term changes to the teat condition on cow level (STC, n; %), stratified by teat-end shape (TES) from 384 milking observations of 129 cows.

|  | STIM | | | |  | LAG | | | |  | STC | |
| --- | --- | --- | --- | --- | --- | --- | --- | --- | --- | --- | --- | --- |
| Item | Mean | SD | Median | Range |  | Mean | SD | Median | Range |  | n | % |
| TES |  |  |  |  |  |  |  |  |  |  |  |  |
| Pointed | 11 | 4 | 10 | 6-23 |  | 74 | 22 | 70 | 20-139 |  | 38/54 | 70 |
| Flat | 11 | 4 | 10 | 5-21 |  | 77 | 17 | 73 | 55-118 |  | 12/33 | 36 |
| Round | 11 | 4 | 10 | 4-28 |  | 73 | 19 | 68 | 29-162 |  | 172/297 | 58 |
