# Supplementary material for: A longitudinal prospective cohort study investigating the association of premilking stimulation and teat-end shape on milking characteristics and teat tissue condition in dairy cows
Source: BMC Vet Res. 2019 Feb 12;15:58. doi: 10.1186/s12917-019-1803-2 (PMC6373114; doi:10.1186/s12917-019-1803-2)
Supplement: Supplementary file 3 — Tabel S3-S6. General linear mixed model results describing the factors associated with total milk yield, two-minute milk yield, milking unit-on time, and time in low milk flow rate from 384 milking observations of 129 cows. (DOCX 19 kb) [file 12917_2019_1803_MOESM3_ESM.docx]

**Supplementary Table 3.** Multivariable general linear mixed model describing the factors associated with total milk yield (kg) from 384 milking observations of 129 cows.

| Item | β^1^ (SE) | *p* | LSM^2^ (SE) |
| --- | --- | --- | --- |
| Intercept | 13.3 (0.5) | <0.001 | – |
| Milking session |  | <0.001 |  |
| Milking #1 | 1.5 (0.2)^a^ |  | 15.7 (0.3) |
| Milking #2 | -2.5 (0.2)^b^ |  | 11.6 (0.3) |
| Milking #3 | -Referent-^c^ |  | 14.1 (0.3) |
| Parity |  | <0.001 |  |
| 1^st^ | -2.3 (0.6)^a^ |  | 12.1 (0.4) |
| 2^nd^ | 0.6 (0.7)^b^ |  | 15.0 (0.5) |
| ≥3^rd^ | -Referent-^b^ |  | 14.4 (0.4) |
| DIM^3^ |  | <0.001 |  |
| ≤100 | 2.3 (0.6)^a^ |  | 14.7 (0.4) |
| 101-200 | 2.0 (0.6)^a^ |  | 14.4 (0.5) |
| >200 | -Referent-^b^ |  | 12.4 (0.5) |

^a-c^Main effects marked with different superscript letters differ at a level of *p* ≤ 0.05 in Tukey-Kramer´s post hoc test.

^1^Linear regression coefficient.

^2^Least squares means in kg.

^3^Stage of lactation (days in milk).

**Supplementary Table 4.** Multivariable general linear mixed model describing the factors associated with two-minute milk yield (kg) from 384 milking observations of 129 cows.

| Item | β^1^ (SE) | *p* | LSM^2^ (SE) | |
| --- | --- | --- | --- | --- |
| Intercept | 4.2 (0.6) | <0.001 | | – |
| Milking session |  | <0.001 | |  |
| Milking #1 | -0.6 (0.1)^a^ |  | | 6.0 (0.3) |
| Milking #2 | -0.6 (0.2)^a^ |  | | 6.0 (0.3) |
| Milking #3 | -Referent-^b^ |  | | 6.5 (0.3) |
| TMY^3^ | 0.2 (0.03) | <0.001 | | – |
| TES^4^ |  | 0.6 | |  |
| Pointed | 0.4 (0.8) |  | | – |
| Flat | -1.1 (1.1) |  | | – |
| Round | -Referent- |  | | – |
| LAG^5^ | -0.008 (0.04) | 0.8 | | – |
| TES × LAG |  | 0.003 | |  |
| Pointed × LAG | -0.2 (0.1)^a^ |  | | 5.1 (0.5) |
| Flat × LAG | 0.2 (0.1)^a^ |  | | 6.8 (0.6) |
| Round × LAG | -Referent-^b^ |  | | 6.5 (0.2) |

^a-b^Main effects marked with different superscript letters differ at a level of *p* ≤ 0.05 in Tukey-Kramer´s post hoc test.

^1^Linear regression coefficient.

^2^Least squares means in kg. The results are averaged over the levels of all other categorical variables included in the model and the mean lag time of 74 s.

^3^Total milk yield (kg).

^4^Teat-end shape. Classified as follows: pointed = 2 or more pointed teats, flat = 2 or more flat teats, round = 3 or 4 round teats.

^5^Preparation lag time (s, 1-unit = 10 s), time between first teat stripping and milking unit attachment.

**Supplementary Table 5.** Multivariable general linear mixed model describing the factors associated with milking unit-on time (s) from 384 milking observations of 129 cows.

| Item | β^1^ (SE) | *p* | LSM^2^ (SE) |
| --- | --- | --- | --- |
| Intercept | 136 (19) | <0.001 | – |
| Milking session |  | 0.02 |  |
| Milking #1 | -14 (6)^a^ |  | 255 (8) |
| Milking #2 | -12 (4)^ab^ |  | 241 (8) |
| Milking #3 | -Referent-^bc^ |  | 243 (8) |
| TMY^3^ | 10 (1) | <0.001 | – |
| Parity |  | 0.03 |  |
| 1^st^ | -30 (12)^a^ |  | 230 (11) |
| 2^nd^ | -13 (13)^ab^ |  | 248 (12) |
| ≥3^rd^ | -Referent-^b^ |  | 261 (9) |
| TES^4^ |  | 0.02 |  |
| Pointed | 36 (15)^a^ |  | 277 (13) |
| Flat | -21 (18)^b^ |  | 220 (17) |
| Round | -Referent-^b^ |  | 241 (6) |

^a-c^Main effects marked with different superscript letters differ at a level of *p* ≤ 0.05 in Tukey-Kramer´s post hoc test.

^1^Linear regression coefficient.

^2^Least squares means in s.

^3^Total milk yield (kg).

^4^Teat-end shape. Classified as follows: pointed = 2 or more pointed teats, flat = 2 or more flat teats, round = 3 or 4 round teats.

**Supplementary Table 6.** Multivariable general linear mixed model describing the factors associated with time in low milk flow rate (s) from 384 milking observations of 129 cows.

| Item | β^1^ (95% CI) | *p* | LSM^2^ (95% CI) |
| --- | --- | --- | --- |
| Intercept | 36.42 (24.10-55.04) | <0.001 | – |
| Milking session |  | <0.001 |  |
| Milking #1 | 1.20 (1.08-1.35)^a^ |  | 14 (13-17) |
| Milking #2 | 1.29 (1.11-1.50)^a^ |  | 16 (13-18) |
| Milking #3 | -Referent-^b^ |  | 12 (11-14) |
| TMY^3^ | 0.94 (0.92-0.96) | <0.001 | – |
| Parity |  | 0.004 |  |
| 1^st^ | 0.75 (0.63-0.89)^a^ |  | 12 (10-14) |
| 2^nd^ | 0.93 (0.76-1.13)^ab^ |  | 15 (12-17) |
| ≥3^rd^ | -Referent-^b^ |  | 16 (14-18) |
| TES^4^ |  | 0.007 |  |
| Pointed | 0.42 (0.24-0.75) |  | – |
| Flat | 0.56 (0.23-1.32) |  | – |
| Round | -Referent- |  | – |
| LAG^5^ | 0.996 (0.97-1.03) | 0.8 | – |
| TES × LAG |  | <0.001 |  |
| Pointed × LAG | 1.15 (1.08-1.23)^a^ |  | 16 (13-19) |
| Flat × LAG | 1.07 (0.97-1.19)^a^ |  | 13 (10-16) |
| Round × LAG | -Referent-^a^ |  | 14 (12-15) |

^a-b^Main effects marked with different superscript letters differ at a level of *p* ≤ 0.05 in Tukey-Kramer´s post hoc test.

^1^Linear regression coefficient.

^2^Least squares means in s. The results are averaged over the levels of all other categorical variables included in the model and the mean lag time of 74 s.

^3^Total milk yield (kg).

^4^Teat-end shape. Classified as follows: pointed = 2 or more pointed teats, flat = 2 or more flat teats, round = 3 or 4 round teats.

^5^Preparation lag time (s, 1-unit = 10 s); time between first teat stripping and milking unit attachment.
